# Regulation of deoxynucleotide metabolism in cancer: novel mechanisms and therapeutic implications

**DOI:** 10.1186/s12943-015-0446-6

**Published:** 2015-09-29

**Authors:** Rebecca Kohnken, Karthik M. Kodigepalli, Li Wu

**Affiliations:** Center for Retrovirus Research, Department of Veterinary Biosciences, The Ohio State University, 1900 Coffey Road, Columbus, OH 43210 USA; Department of Microbial Infection and Immunity, The Ohio State University, Columbus, OH 43210 USA; Comprehensive Cancer Center, The Ohio State University, Columbus, OH 43210 USA

**Keywords:** Intracellular dNTP, Regulation, Cancer, Cell proliferation, SAMHD1

## Abstract

Regulation of intracellular deoxynucleoside triphosphate (dNTP) pool is critical to genomic stability and cancer development. Imbalanced dNTP pools can lead to enhanced mutagenesis and cell proliferation resulting in cancer development. Therapeutic agents that target dNTP synthesis and metabolism are commonly used in treatment of several types of cancer. Despite several studies, the molecular mechanisms that regulate the intracellular dNTP levels and maintain their homeostasis are not completely understood. The discovery of SAMHD1 as the first mammalian dNTP triphosphohydrolase provided new insight into the mechanisms of dNTP regulation. SAMHD1 maintains the homeostatic dNTP levels that regulate DNA replication and damage repair. Recent progress indicates that gene mutations and epigenetic mechanisms lead to downregulation of SAMHD1 activity or expression in multiple cancers. Impaired SAMHD1 function can cause increased dNTP pool resulting in genomic instability and cell-cycle progression, thereby facilitating cancer cell proliferation. This review summarizes the latest advances in understanding the importance of dNTP metabolism in cancer development and the novel function of SAMHD1 in regulating this process.

## Introduction

Balanced levels of intracellular dNTPs, the building blocks of DNA, are critical in maintaining the genomic integrity of cells. While a reduction or imbalance in dNTPs is known to result in genotoxicity and increased mutagenesis, an increase in dNTPs often results in uncontrolled DNA replication with reduced fidelity that can contribute to cancer development [1, 2]. Indeed during DNA replication, an unequal and abnormal increase in concentrations of specific dNTPs may result in reduced fidelity [1, 2]. Many types of cancer cells demonstrate impairment of intracellular dNTP homeostasis, which supports rapid cellular proliferation, enhanced mutagenesis, and contributes to dysregulation of the cell cycle [3]. Nucleotide metabolism, therefore, is a common therapeutic target used in the treatment of many types of cancer. Nucleoside analogs as well as enzyme inhibitors aim to disrupt the synthetic pathways which result in imbalance of dNTPs in cancer cells [4]. However, the integrated mechanisms, such as anabolic and catabolic pathways, cell cycle control, and DNA repair, which result in impaired dNTP homeostasis in cancer, are incompletely understood.

The intracellular dNTP balance is regulated in part by sterile alpha motif (SAM) and histidine/aspartate (HD)-domain containing protein 1 (SAMHD1), the first dNTP triphosphohydrolase (dNTPase) discovered in mammalian cells [5–8]. SAMHD1 acts as the host restriction factor that inhibits human immunodeficiency virus type 1 (HIV-1) infection by reducing intracellular dNTP levels [9]. Human *SAMHD1* mutations can cause a severe autoimmune disorder [10], suggesting the importance of its dNTPase function in innate immunity. Mutations of *SAMHD1* have been identified in several human cancers [11–19]. SAMHD1 expression is downregulated in many cancers, including leukemia, lymphoma, and solid cancers, such as breast and lung cancer [11, 20, 21]. Restoration of SAMHD1 expression has been reported to reduce cellular proliferation *in vitro* [11, 21]. Based on these recent findings, SAMHD1 is proposed to have anti-proliferative and tumor suppressive functions in several cancers. Since dNTP metabolism and balance is critical in carcinogenesis, the dNTPase activity of SAMHD1 may mediate its tumor suppressive function. Despite several advancements in in the use of therapeutic nucleoside analogs to target dNTP metabolism, there is yet a need to develop novel and more effective therapeutic strategies in cancer treatment. Further studies to understand the physiological significance of SAMHD1 in cancer can aid in this process.

Although numerous studies have investigated the dNTPase function of SAMHD1 in viral restriction and immune responses, its significance in cancer development and progression has lately been an emerging interest. Functional significance of SAMHD1 and its dNTPase activity in cancer pathophysiology has not yet been reviewed. Here we highlight the importance of dNTP homeostasis in cancer and dNTP regulation by SAMHD1. We also discuss the potential role of SAMHD1 as a tumor suppressor and future studies required to better understand its function for cancer therapeutic development.

## Regulation of intracellular dNTPs and its role in cancer

### Intracellular dNTP synthesis and regulation

Coordinated synthesis and degradation of dNTPs resulting in a balanced intracellular dNTP pool is critical for numerous cellular processes, such as fidelity of DNA synthesis and DNA damage repair [22]. Two distinct pathways that synthesize dNTPs are the *de novo* synthesis in the cytoplasm, and the salvage pathway that takes place both in cytoplasm and mitochondria. The rate-limiting step of *de novo* dNTP synthesis is catalyzed by ribonucleotide reductase (RNR) that converts ribonucleotide diphosphates to deoxyribonucleotides [3]. Degradation of dNTPs as part of the salvage pathway is accomplished by deaminases and phosphorylases, as well as the mammalian triphosphohydrolase, SAMHD1 [7, 8]. Optimization of dNTP pools is achieved by cell cycle-dependent activity and allosteric regulation of RNR and SAMHD1 [23]. Actively proliferating cells have an approximately 10-fold higher dNTP pool than quiescent cells that are in G_0_/G_1_ phase [24]. The dNTP pool is greatly expanded during G_1_ to S-phase transition, and remains abundant until DNA synthesis is complete [24]. This biphasic regulation is critical to supply dNTPs for DNA synthesis, and to prevent excess intracellular dNTPs in the absence of DNA replication, which can contribute to innate immune activation [25] and cancer development [22].

### Dysregulation of dNTP in cancer development

The complement of intracellular dNTPs has numerous implications for DNA replication, mutagenesis, DNA repair, and therefore in cancer development. Recent progress in literature suggests that RNR-mediated increase in dNTP pools is accompanied by higher mutation rates due to reduced fidelity of DNA replication or activation of translesion synthesis [26]. These studies suggest that increased dNTP pools upon altered RNR activity may cause increased mutation rates. However, it is important to note that altered RNR activity can also affect its function in DNA repair (25). Therefore, further studies are required to rule out the possibility of altered DNA repair functions of RNR leading to increased mutation rate. Indeed, dNTP pools are generally greater in transformed cell lines compared to normal cells [27]. Mutator phenotypes are characterized by increased somatic mutation frequency in pre-cancerous cells that accounts for high number of mutations in cancer cells, consistent with what is observed with dNTP pool imbalances [24, 28, 29]. These pre-cancerous cells are characterized by enhanced mutagenesis, stimulation of genetic recombination, increased frequency of chromosomal abnormalities, DNA strand breaks and cell death [26]. Imbalance in cellular dNTP pool causes a hypermutator phenotype, associated with DNA replication stress and altered replication fork velocity [3, 11]. Mechanisms of mutagenesis conferred by imbalanced dNTP pool are not fully understood, but likely include nucleotide misinsertion during DNA replication, indirect inhibition of proof-reading, or forced frameshift mutations [26].

In response to DNA damage, dNTP levels in S-phase increase approximately by 4-fold [30]. Both RNR and SAMHD1 can be recruited to sites of DNA damage to tightly regulate the dNTPs supplied for DNA repair machinery [11, 30]. Replication stress from increased mutation rate contributes to genomic instability and activates the DNA damage response, which then may further exacerbate mutagenesis by altering the balance of the dNTP pool [31, 32]. It is interesting to note that pre-cancerous cells show evidence of increased DNA damage response activation [31, 32]. Together, these studies suggest that in cells with elevated activation of DNA damage responses, increased dNTP levels may lead to malignancy.

Nucleotide metabolism plays an important role in senescence and autophagy in cancer cells. Senescence may occur as a tumor suppressive mechanism early on in tumor initiation, while autophagy is a common mechanism that tumor cells use to survive under metabolic stress [3, 33]. Nucleotide metabolism therefore has implications in genomic instability and mutation as part of tumor initiation, and resistance to apoptosis during tumor promotion, two of the important hallmarks of cancer development. Thus, regulators of dNTP pool are important targets of cancer therapy.

### Current cancer therapy targeting dNTP metabolism

**Fig. 1:**
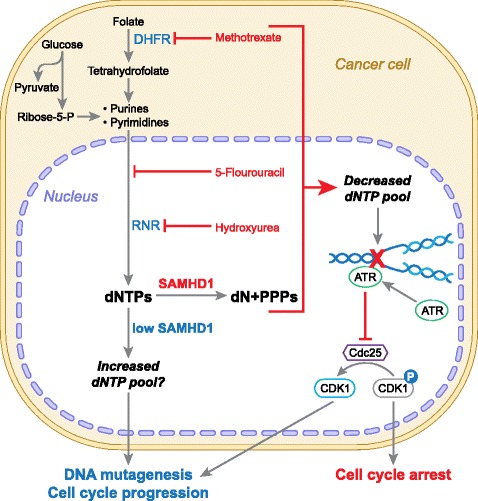
Dysregulation of dNTPs in cancer pathogenesis and its targeted therapy. Nucleotides are derived from multiple intracellular sources, including products of glycolysis, folate cycle, and scavenging of degraded components. The reduction of dihydrofolate to active tetrahydrofolate is inhibited by the chemotherapeutic methotrexate. Pyrimidine and purine bases are both reduced to deoxynucleosides (dN) by ribonucleotide reductase (RNR). This reaction is inhibited by the chemotherapeutic hydroxyurea. Other steps in this reaction are inhibited by numerous nucleoside analogs (“antimetabolite” compounds) including 5-fluorouracil. These drugs function by limiting the deoxynucleoside triphosphate (dNTP) pool available for DNA synthesis and triggering the S-phase checkpoint via the action of ATR and Chk1, resulting in cell cycle arrest by inhibiting the activation of cyclin dependent kinase 1 (CDK1). A potentially critical regulator of this pathway is SAMHD1, which hydrolyses dNTPs into products that are then recycled or degraded. By this action, SAMHD1 limits dNTP pool in G1 phase and prevents DNA replication. With loss of function or repression of SAMHD1 expression, the dNTP pool is not reduced which can result in DNA damage and inappropriate cell cycle progression. DHFR, dihydrofolate reductase; PPPs, triphosphate; ATR, ataxia-telangiectasia and Rad3-related protein; Cdc25, cell division cycle 25

Given the important role of nucleotide metabolism in cell proliferation, transformation and tumor progression, inhibition of nucleotide synthesis has been commonly used in treatment of cancer, as well as infectious and immune-mediated diseases [3]. Inhibiting DNA precursor synthesis or incorporation of nucleoside analogs into DNA results in DNA damage and stalled replication forks, followed by activation of the S-phase checkpoint, which may lead to cell death by apoptosis [3] (Fig. 1). Methotrexate and similar compounds that inhibit dihydrofolate reductase required for purine biosynthesis [34], are widely used in chemotherapy for solid and lymphocytic tumors. On the other hand, purine and pyrimidine analogs such as forodesine and gemcitabine respectively, can disrupt DNA replication [35]. Moreover, 5-fluorouracil is a pyrimidine adduct that is misincorporated into DNA and inhibits nucleotide synthesis [34]. Most of clinically available nucleoside analogs act by incorporation into DNA, which relies on balanced dNTP pool for high fidelity of incorporation during replication. Excess intracellular dNTPs may out-compete these nucleoside analogs, thus conferring chemotherapeutic resistance [36]. For this reason, these drugs may be used in combination with other therapies, such as inhibitors of RNR that reduce dNTP pool. Efficient RNR inhibition is accomplished by hydroxyurea, a free radical scavenger and iron chelator that inactivates the catalytic capacity of the R2 subunit of RNR [3]. Current clinically used inhibitors of RNR, such as hydroxyurea or gemcitabine, have limitations, including severe cytotoxicity, short half-life, and drug resistance when used as a single therapy. New horizons for nucleotide synthesis-directed therapy include small molecule targeting subunit activity modulation, such as anti-sense oligonucleotides and gene therapy targeting RNR [3, 26].

It is currently unclear whether SAMHD1 can degrade these nucleoside analogs in cells and reduce their anti-cancer potency. A study indicates that reduction of SAMHD1 levels in T-cells significantly decreases HIV-1 sensitivity to thymidine analogs (such as zidovudine or stavudine), but not other tested nucleotide analogs of HIV-1 reverse transcriptase inhibitors [37]. These results suggest that SAMHD1 may have a differential effect over the different dNTPs. Owing to its dNTPase activity, SAMHD1 may serve as an intriguing target of cancer therapy as reduction of dNTP pool may prevent tumorigenesis or an increase in efficacy of anti-nucleotide chemotherapeutics (Fig. 1).

## Regulation of nucleotide metabolism by dNTP hydrolysis

### Regulating dNTP homeostasis

SAMHD1 was identified in human myeloid cells as a novel HIV-1 restriction factor [5, 6]. SAMHD1 is responsible for maintaining low levels of intracellular dNTPs in non-dividing cells [9, 38, 39]. In non-transformed fibroblasts, knockdown of SAMHD1 results in loss of cell-cycle regulation, and cells accumulate in G_1_ with oversized dNTP pool [40]. Therefore, SAMHD1 influences cell cycle progression and DNA replication by degrading dNTPs [40] (Fig. 1). SAMHD1 siRNA-mediated knockdown has differential effects on the dNTP pools of proliferating cells and quiescent cells [40]. In proliferating lung or skin fibroblasts, siRNA-mediated knockdown of SAMHD1 leads to loss of the cell-cycle regulation of dNTP pool sizes and thus resulting in dNTP imbalance [40]. On the other hand, in cells made quiescent via serum starvation, SAMHD1 down-regulation leads to a marked expansion of dNTP pools [40]. The function of SAMHD1 in sensing intracellular dNTPs is critical for maintaining pool balance in cells at the appropriate phase in cell cycle.

The opposite role of increasing the intracellular dNTP pool is achieved by RNR, which catalyzes the rate-limiting step in *de novo* nucleotide synthesis by converting ribonucleotides to deoxyribonucleotides [3, 24]. RNR heterotetramer is present at consistent levels throughout the cell cycle and functions in *de novo* nucleotide synthesis even in non-dividing cells [27]. The gene encoding the active subunit, R1, is transcriptionally regulated, responding to DNA replication stress and progression through the cell cycle [31]. RNR transcription increases by 10–20 fold in S phase over the levels in G1 phase [27, 31]. RNR can also be recruited to sites of DNA damage, by the virtue of p53-inducible subunit R2, to supply the necessary dNTPs for the damage repair machinery [27]. Cancer cells require RNR for *de novo* synthesis of dNTPs and elevated RNR expression is a characteristic of many cancers, which contribute to increased dNTP levels and uncontrolled cellular proliferation [27].

Both SAMHD1 and RNR have similar regulation mechanisms of their expression and activity, which are cell-cycle dependent and tightly controlled in cells. Both enzymes are active oligomers with two types of allosteric sites that sense the concentration of nucleotides in the cell and control enzymatic activity [23, 24, 41]. Regulation of SAMHD1 dNTPase activity is achieved by allosteric binding of dGTP or GTP to the first allosteric site, followed by binding of any dNTP to the second allosteric site, causing a conformational change in the substrate-binding pocket [23, 42]. SAMHD1 has sensory activity that detects the cellular dNTP pool composition, and allows a feedback system to achieve a balance with the opposite catalytic activity of RNR [23, 41]. The most sensitive regulator of dNTP pool is likely dATP, as it has higher affinity for the second allosteric site of SAMHD1, weak affinity for the catalytic site of SAMHD1, and is produced least efficiently by RNR [26, 43]. The dATP:ATP ratio is likewise an important regulator of RNR activity [26]. Elevations in dATP concentrations are inhibitory to the anabolic activity of RNR, whereas increased ATP concentration is stimulatory [26]. Therefore, dATP plays a fine-tuned regulatory role in providing negative feedback from SAMHD1 to RNR and vice versa [23, 26, 41, 43]. Allosteric binding of dNTPs is responsible for tetramerization of SAMHD1 to its functional catalytic state. Tetramer-disrupting mutations abolish dNTPase activity as well as the ability of SAMHD1 to restrict HIV-1 infection [23, 42].

### Restricting viral infection

SAMHD1 inhibits retroviral replication in non-dividing cells by depleting the intracellular dNTP pool to a level that is limiting for viral reverse transcription [9, 38, 39]. In terminally differentiated macrophages and dendritic cells, or resting CD4^+^ T-cells with arrested cell cycle (G_0_/G_1_), dNTP levels are significantly decreased by SAMHD1, and therefore HIV-1 infection is restricted [9, 38, 39]. Vpx, a protein encoded by HIV-2 or certain simian immunodeficiency viruses, causes proteasomal degradation of SAMHD1, which is associated with an increase in dNTP pool, accelerated proviral DNA synthesis, and enhanced viral infectivity [9, 38, 39]. Low cellular dNTP serves as a common mechanism of SAMHD1-mediated retroviral restriction. In addition to HIV-1, SAMHD1 also restricts many other retroviruses and DNA viruses (such as herpes simplex virus 1 and hepatitis B virus) in non-dividing cells [44–46]. However, it remains unclear how these viral restriction studies provide any clues that may link with cell cycle dysregulation in cancer cells, particularly in the context of viral infection.

### Interaction with cell-cycle regulatory proteins

Phosphorylation at threonine (T) 592 by cyclin dependent kinases (CDK) regulates dNTPase activity and HIV-1 restriction activity of SAMHD1 [47–49]. CDK1 as well as CDK2 in complex with cyclin A can bind to and phosphorylate this site [25, 47–49]. CDK2 interaction with SAMHD1 is regulated by CDK6 in T-cells and macrophages [50]. Type I IFN reduces phosphorylation of SAMHD1 at the T592 residue [47], likely by inducing CDK inhibitors, such as p21, resulting in HIV-1 restriction and decreased dNTP pool [50, 51]. For proper maintenance of dNTP levels, SAMHD1 protein must be reduced in the cell during S-phase to facilitate DNA replication [40]. In differentiated human macrophages, SAMHD1 interacts with cyclin L2, forming an ubiquitin ligase complex and resulting in its proteasomal degradation [52]. However, it is unclear whether the degradation process of endogenous SAMHD1 occurs in other cell types or in dividing cells. Phosphorylation of SAMHD1 by CDKs may be the signal for its proteasomal degradation in a cell type- or cell cycle-dependent manner. With an understanding of SAMHD1 dNTPase function and dNTP regulation in cancer, we discuss the physiological consequences of alterations in SAMHD1 expression.

## SAMHD1 alterations in an autoimmune disease and cancers

### Mutations of SAMHD1 in an autoimmune disease

SAMHD1 is a negative regulator of IFN-induced innate immune responses [53]. Aicardi-Goutieres syndrome (AGS) is an hereditary neurodegenerative autoimmune disorder that is characterized by progressive encephalopathy and is accompanied by increased IFN-α production [54]. AGS is attributed to defective clearance of excessive intracellular (self) nucleic acids, which trigger immune responses resulting in activation of IFN pathway in a manner that mimics viral infection [10, 55]. *SAMHD1* homozygous mutations have been identified in 17 % of patients with AGS, suggesting a pivotal role of SAMHD1 in preventing activation of innate immune response to self-nucleic acids [10]. Further, a homozygous deletion (~9 Kb) in the *SAMHD1* gene was identified in patients with atypical AGS, characterized by genomic instability [56]. Mutations in *SAMHD1* result in increased dNTP pools in fibroblasts from AGS patients [25], suggesting that the dNTPase function of SAMHD1 prevents autoimmunity by maintaining genome stability. Together, these studies implicate SAMHD1 in regulation of self-nucleic acid stimulated-autoimmune responses.

### Mutations of SAMHD1 in cancers

One of the most critical mechanisms by which cancer cells grow uncontrollably is by inhibiting the function of tumor suppressor genes [57]. This is achieved mainly through mutation (leading to loss of function) and/or downregulation of tumor suppressor genes that normally arrest cell growth [57]. Studies using CLL patient samples have identified *SAMHD1* mutations in 4 out of 160 cases (2.5 %) [13]. Acquired *SAMHD1* mutations were reported at a frequency of 11 % in relapsed or chemotherapy refractory CLL patients and 3 % in the pretreatment group [11]. These findings suggest that SAMHD1 mutation can be an important driving factor contributing to CLL progression, and can potentially be utilized as a biomarker for CLL prognosis.

*SAMHD1* somatic mutations have been identified in several human cancers, including chronic lymphocytic leukemia (CLL) [11, 13, 16], myeloma [18], breast cancer [17], lung carcinoma [14], colon and rectal cancer [17, 19, 58], pancreatic cancer [12], and glioblastoma [15]. All the identified SAMHD1 alterations in cancer are summarized in Table 1. Of note, several of these mutations are located in the catalytic region of HD domain of SAMHD1 that is responsible for its dNTPase activity (Table 1) [7, 8]. However, it is important to note that it is still unknown whether these specific mutations in the HD domain of SAMHD1 can directly affect its dNTPase function. Generation and characterization of SAMHD1 variants harboring these specific mutations can help define their activity changes. Importantly, physiological significance and functional contributions of these *SAMHD1* mutations to progression of cancer also needs to be investigated, especially since the *SAMHD1* knockout in mice does not result in spontaneous tumorigenesis [53, 59]. Further functional analyses of SAMHD1 mutations identified in human cancer cells would aid in answering these important questions.

**Table 1:** Summary of the identified SAMHD1 alterations in various human cancers

Cancers	Identified *SAMHD1* alterations				References
	Gene mutations		Epigenetic alterations	Consequences	
	Frequencies	Amino acid changes (domain locations)^a^, ^b^			
Breast cancer	0.4 %^c^ (4/981 cases)	N.A.	N.A.	Reduced SAMHD1 protein	[11, 17]
Chronic lymphocytic leukemia	2.5 %^a^ [13] (4/160 cases)	N.A.	N.A.	Reduced SAMHD1 mRNA and protein	[11, 13, 16]
Colorectal cancer	2.2 %^c^ (5/223 cases)	F59C (SAM domain)^d^ D207Y (HD domain)^d^ R226H (HD domain)^d^ T232M (HD domain)^d^ S247Y (HD domain)^d^ K288T (HD domain)^d^	N.A.	N.A.	[17, 19, 58]
Cutaneous T-cell lymphoma	N.A.	N.A.	Promoter DNA methylation	Reduced SAMHD1 mRNA and protein	[20]
Glioblastoma	0.3 %^c^ (1/290 cases)	N.A.	N.A.	N.A.	[15]
Lung cancer	1.7 %^c^ (4/228 cases)	A441T (HD domain)^d^	Promoter DNA methylation	Reduced SAMHD1 mRNA and protein	[14, 21]
Myeloma	1 %^c^ (2/205 cases)	Y521D (HD domain)^d^	N.A.	N.A.	[18]
Pancreatic cancer	1.1 %^c^ (1/91 cases)	N.A.	N.A.	N.A.	[12]

*N.A.* Information not available

^a^The mutation details are based on cited literature

^b^It is unclear whether all of these mutations in the HD domain of SAMHD1 can directly affect its dNTPase function

^c^The mutation rates are based on TCGA data analysis via cBioportal (http://www.cbioportal.org/public-portal/) [67]

^d^The amino acid (aa) changes and their positions; the sterile alpha motif (SAM) domain (aa 45-110); the catalytic region (aa 167-311) of the histidine-aspartate (HD) domain (aa 115-562) [68]

### Downregulation of SAMHD1 expression in cancers

*SAMHD1* mRNA levels were significantly lower in CLL patient B-cells with *SAMHD1* mutation relative to normal B-cells [11]. Although this study suggests that these mutations result in reduction of SAMHD1 mRNA levels, the exact mechanism involved in this process is unknown. It is important to determine whether these mutations lead to reduced transcription of SAMHD1 or if they cause a post-transcriptional change such as nonsense-mediated decay of *SAMHD1* mRNA. SAMHD1 expression is also significantly lower in other cancers, including cutaneous T-cell lymphoma (CTCL) [20], lung carcinoma [21], breast carcinomas and various tumor cell lines [11].

CTCL is a subset of non-Hodgkin’s lymphomas that is characterized by infiltration and proliferation of malignant CD4^+^ T-lymphocytes into the skin [60]. Decreased mRNA and protein levels of SAMHD1 were identified in peripheral blood mononuclear cells (PBMCs) of CTCL patients relative to healthy donors [20]. However, it is unknown whether decreased SAMHD1 expression has an effect on CTCL lymphomagenesis, if restored SAMHD1 expression can rescue CTCL phenotype, or whether SAMHD1 mRNA/protein expression correlates with patient prognosis. Lung carcinoma patients have reduced SAMHD1 mRNA and protein [21], but a direct correlation to disease progression or outcome has not been established. Overexpression of SAMHD1 in a lung cancer cell line results in decreased proliferation *in vitro,* with a concomitant increase in intracellular dNTP levels [21]. Similarly, overexpression of SAMHD1, but not the dNTPase-defective mutant, can significantly reduce HeLa cell proliferation [11].

### Epigenetic regulation of SAMHD1 in cancers

Epigenetic mechanisms play a major role in cancer development and progression by modulating gene expression [61]. DNA methylation and chromatin remodeling via histone acetylation or deacetylation are the most important epigenetic mechanisms through which cancer cells achieve increased expression of oncogenes or decreased expression of tumor suppressor genes [61]. Thus, many therapeutic strategies target epigenetic mechanisms in cancer [62].

Studies in lung cancer and CTCL patients have revealed the role of epigenetic mechanisms in downregulation of SAMHD1 expression. The *SAMHD1* promoter in PBMCs of CTCL patients is highly methylated, while it is unmethylated in PBMCs from healthy donors [20]. This was also observed in human lymphoma/leukemia CD4^+^ T-cell lines relative to primary CD4^+^ T-cells that have high endogenous SAMHD1 expression [63]. Importantly, treatment with DNA methyltransferase inhibitors in CD4^+^ T-cell lines rescues SAMHD1 mRNA and protein expression, validating that DNA methylation has a direct inhibitory effect on SAMHD1 transcription [63]. Similar effects of SAMHD1 promoter methylation on its expression were identified in lung cancer tissues [21]. Interestingly, inhibition of histone deacetylation leads to induction of SAMHD1 mRNA and protein levels in CD4^+^ T-cells, suggesting that histone deacetylation also contributes to repression of SAMHD1 expression [63]. Despite implications for epigenetic downregulation of SAMHD1 in cancer, the exact mechanisms are not known. Better understanding of these pathways may aid in development of therapeutic strategies targeting epigenetic modifications.

**Fig. 2:**
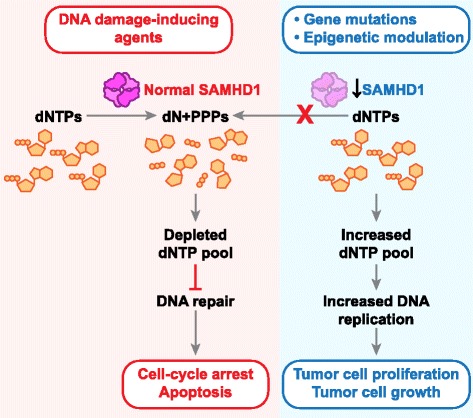
SAMHD1 may function as a potential tumor suppressor via regulation of dNTPs. SAMHD1 is a novel mammalian dNTP triphosphohydrolase enzyme that helps in maintenance of intracellular dNTP homeostasis. SAMHD1 has been identified to be downregulated in cutaneous T-cell lymphoma and lung cancer patient samples via increased promoter DNA methylation. Downregulated SAMHD1 expression may increase the dNTP pool in these cancers resulting in enhanced DNA replication and thus tumor cell growth and proliferation. On the other hand, overexpression of SAMHD1 protein upon treatment with DNA damage-inducing agents may result in depleted dNTP pools leading to defective DNA damage repair mechanisms and thus cell cycle arrest and apoptosis

Gene expression is also regulated by microRNAs and transcription factors. Although, transcription factors that can regulate SAMHD1 expression are yet to be identified, one study has indicated that microRNA-181 binds directly to the 3’ untranslated region of *SAMHD1* mRNA and downregulates its expression [64]. Overexpression of microRNA-181 in monocytic THP-1 cells reduced endogenous SAMHD1 mRNA and protein levels, while microRNA-181 downregulation in T-cell leukemic-derived Jurkat cells lead to an increase in SAMHD1 mRNA and protein [64]. It is unclear whether microRNA-181 downregulates SAMHD1 expression in cancer patients. Together these findings support the hypothesis that SAMHD1, through its dNTPase function, could potentially act as a tumor suppressor (Fig. 2).

## SAMHD1 may act as a tumor suppressor by maintaining genome stability

Cancer cells frequently exhibit genomic instability and are characterized by increased mutation rate, increased incidence of chromosomal rearrangement, and frequent retroelement insertions. Many studies demonstrate that dNTP homeostasis is necessary for maintenance of accurate DNA replication and efficient DNA damage repair [22]. By regulating dNTP homeostasis, SAMHD1 expression has implications in cancer.

SAMHD1 is expressed ubiquitously in undifferentiated and differentiated cell types to regulate DNA damage signaling and facilitate proper activation of the innate immune response [25]. Constitutive DNA damage signaling, as noted in pre-neoplastic cells with genomic instability, is associated with cell cycle delay, senescence, and upregulation of IFN-stimulated genes. AGS patient cells have increased sensitivity to genotoxic stimuli, and respond to DNA damage with increased rates of mutagenesis [10]. However, it remains unclear whether AGS patients with *SAMHD1* mutations have a higher predisposition to cancer development. Notably, an AGS patient harboring a germ line mutation in *SAMHD1* developed CLL [11]. Loss of SAMHD1 enhances resistance to chemotherapy-induced DNA damage [11]. Following treatment with DNA-damaging agents, SAMHD1 protein is recruited to sites of double-stranded DNA breaks and results in increased cell death, a protective mechanism in cells [11]. In addition to a causal link between DNA damage signaling and innate immune activation, SAMHD1 also prevents aberrant synthesis of DNA species and accumulation of endogenous retroelements that can activate DNA sensors and trigger type I IFN immune responses [10, 25, 53].

Approximately 17 % of the human genome is composed of retrotransposons that may cause genomic insertions, termed long interspersed elements (LINE) [65]. SAMHD1-mediated LINE1 inhibition is through blocking of the reverse transcriptase activity of the element. Moreover, AGS-associated SAMHD1 mutants are defective in inhibiting LINE1 and silencing SAMHD1 in cells results in an increase in retrotransposition activity [65]. Thus, SAMHD1 may play a key role in reducing random insertions by retroelements that contribute to genomic instability and lead to cancer development.

A homozygous large deletion in SAMHD1 was detected in patients with atypical AGS featuring multiple mitochondrial DNA deletions [56], highlighting the importance of balanced cytoplasmic dNTP pool affecting mitochondrial pools [40, 56]. Concentrations of dNTPs are highly correlated between cytoplasm and mitochondria in non-transformed cells [27]. The dNTP levels in both cytoplasm and mitochondria are critical to fidelity of DNA replication, mutagenesis, and genome integrity [27, 66]. Thus, loss of SAMHD1 activity has functional consequences for dysregulated DNA replication, damage repair, and genomic integrity, thereby contributing to tumorigenesis.

## Conclusions and perspectives

Mutagenesis and disruption of genomic stability are two important factors leading to cancer development, which can be prevented by maintenance of optimal intracellular dNTP pools. Balanced dNTP levels are required for DNA damage repair that is necessary to prevent cancer initiation [22]. Excess intracellular dNTP pool is common in cancer cells, not only conferring proliferative ability to the cell, but also posing a challenge for successful therapy targeting dNTP metabolism. Nucleoside analogs have been a mainstay of cancer treatment for decades [4], however persistently high levels of dNTP in the cell can out-compete the drug and result in cancer resistance. Mutations in SAMHD1 have been suggested to contribute to therapeutic resistance due to a loss of dNTP hydrolysis [11]. Combination therapies that target both the synthetic and hydrolytic pathways of nucleotides would likely result in better outcomes. Additionally, restoration of SAMHD1 by targeting the epigenetic mechanisms using histone deacetylase inhibitors may sensitize cancer cells to DNA damage-inducing therapies, such as treatment with poly (ADP ribose)-polymerase inhibitors. Therefore, it will be important to invest further efforts in understanding the complexities of nucleotide metabolism as it relates to regulation of cell cycle, mutagenesis, and cancer development.

SAMHD1 is a key regulator of dNTP homeostasis via its dNTPase function [7, 8] and this activity is important to prevent innate and autoimmune responses [7]. Similarly, dNTPase activity of SAMHD1 could play an anti-proliferative role in cancer pathophysiology (Fig. 2). Several studies in different cancers demonstrated SAMHD1 mutation or downregulation, implicating it as a potential tumor suppressor. Restoring SAMHD1 expression in cancer cell lines leads to decreased cell proliferation supporting the hypothesis that SAMHD1 has anti-proliferative effects in transformed cells [11, 21]. Additionally, SAMHD1 may assist in DNA damage response and contribute to cell cycle regulation (Fig. 2).

Despite rapid progress of structural and functional studies of SAMHD1, its exact role and mechanisms by which it regulates cancer cell growth and proliferation remain to be understood further. *In vivo* studies of loss of function and expression of SAMHD1 in cancer are lacking. Although mouse models with *SAMHD1* knockout have been established [53, 59], these mice do not develop spontaneous cancers at the age of 70–96 weeks [53, 59], indicating that SAMHD1 reduction alone is not sufficient to develop cancer in mice. As with most tumor suppressor genes, it is likely that additional hits are required. Loss or impaired SAMHD1 function could confer partial but significant selective advantage to proliferating cancer cells due to an increase or imbalance in intracellular dNTPs (Fig. 2).

Understanding the mechanisms that downregulate SAMHD1 in cancers is of utmost importance as it could support developing novel strategies to restore SAMHD1 expression in cancer cells and thus reduce tumorigenicity. Epigenetic mechanisms regulate SAMHD1 expression in CTCL and lung cancers [20, 21]. Identifying the detailed mechanisms and molecules that are involved would lead to approaches that restore SAMHD1 to physiologically normal levels. This may enhance the efficacy of the current DNA damage-inducing strategies in cancer treatment that target dNTP regulation. In conclusion, illumination of the significance of SAMHD1 in cancer development would open up new avenues of discovery in studying dNTP regulation and cancer biology.

## Abbreviations

AGS: Aicardi-Goutieres syndrome
CDK: Cyclin-dependent kinase
CLL: Chronic lymphocytic leukemia
CTCL: Cutaneous T cell lymphoma
dNTP: Deoxynucleotide-triphosphate
GTP: Guanosine triphosphate
HIV: Human immunodeficiency virus
IFN: Interferon
LINE: Long interspersed elements
RNR: Ribonucleotide reductase
SAMHD1: Sterile alpha motif and HD-domain containing protein 1
Vpx: Viral protein X
